# A Unique Case of Popliteal Artery Thrombosis in Isolated Prothrombin Gene Mutation

**DOI:** 10.7759/cureus.12376

**Published:** 2020-12-30

**Authors:** Sakthivelavan Duraipandian-Sendiladibban, Kathleen Hoban, Sumathilatha Sakthi-Velavan, Ramesh Adhikari

**Affiliations:** 1 Hospital Medicine, Franciscan Health, Lafayette, USA; 2 Biomedical Sciences, Marian University College of Osteopathic Medicine, Indianapolis, USA; 3 Geriatrics, Brown University, Providence, USA

**Keywords:** prothrombin gene mutation, ptgm, popliteal artery thrombosis, venoud thromboembolism, thrombophilia

## Abstract

A prothrombin gene mutation (PTGM) is the second common cause of inherited thrombophilia after factor V Leiden. Hypercoagulable conditions have traditionally been reported to cause venous thrombosis, while arterial thrombosis is a rare occurrence. Studies have reported cases of preexisting hypercoagulable conditions associated with PTGM presenting as thromboembolism; however, none have been recorded with isolated PTGM. A 55-year-old patient was diagnosed to have unilateral popliteal artery thrombosis. He had a past history of provoked deep vein thrombosis. Investigations confirmed PTGM, and no other associated hypercoagulable conditions or peripheral vascular disease were identified. Embolic sources from the heart, aorta, and an atrial septal defect were ruled out. The patient responded to heparin infusion and catheter-directed thrombolysis using TPA. The case is being reported for its uniqueness since this is the first documented case of popliteal artery thrombosis in a patient with isolated PTGM.

## Introduction

A prothrombin gene mutation (PTGM) results in gain of function and the ensuing hypercoagulability is the second common cause of inherited thrombophilia after Factor V Leiden. Although venous thromboembolism is known to be common among hypercoagulable conditions, arterial thromboembolic events are rare. A 2003 meta-analysis reported only a slight association (OR 1.32; 95% CI 1.03-1.69) between PTGM and arterial thrombotic events (myocardial infarction, ischemic stroke, or peripheral vascular disease) [1]. This report aims at presenting the first case of popliteal artery thrombosis in a patient with isolated PTGM.

## Case presentation

**Figure 1:**
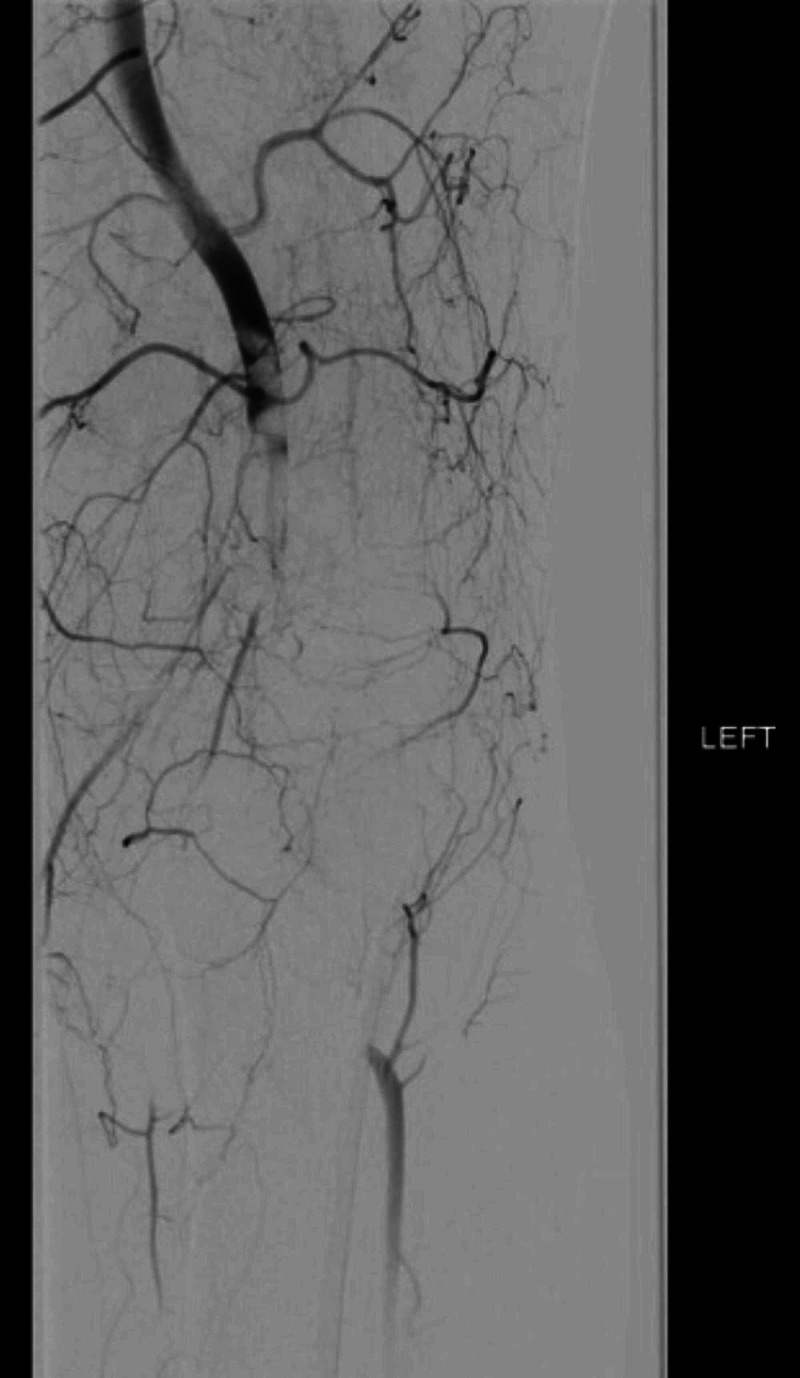
Angiogram showing left popliteal artery occlusion

**Figure 2:**
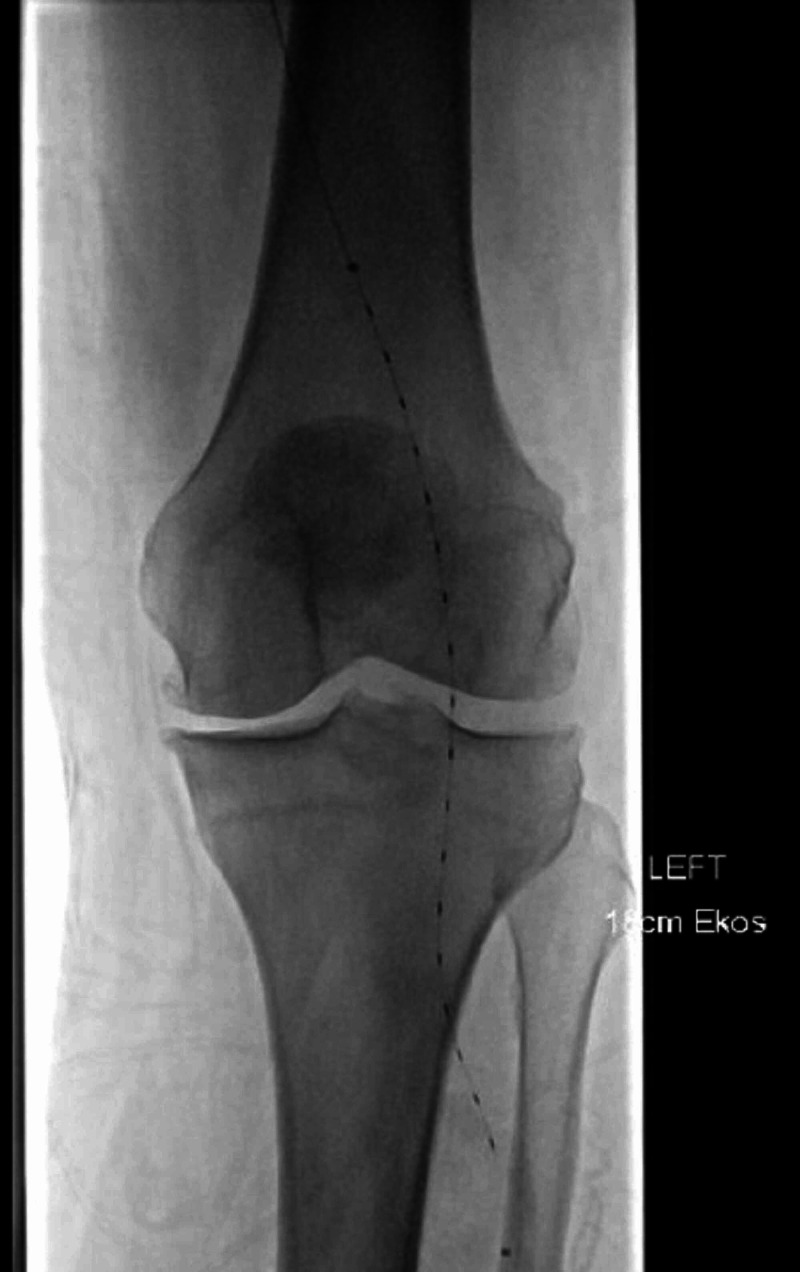
Thrombolytic catheter in the left popliteal artery

A 55-year-old gentleman with past medical history of a provoked DVT in the left leg post ankle fracture two years ago and being off anticoagulation for last one year presented with complaints of sudden onset of left leg pain and numbness. An arterial duplex revealed total occlusion of left popliteal artery. Venous dopplers confirmed extensive bilateral lower extremity DVT also. Patient was treated with heparin infusion and findings on angiogram are shown in figure 1. He underwent ultrasonic catheter directed thrombolysis with TPA (figure 2); post-operatively, his symptoms resolved. An investigation into the etiology of the thrombus disclosed a heterozygous PTGM and no other hypercoagulable conditions were identified. He had no history of peripheral vascular disease. Embolic sources from heart, PFO and an aortic thrombus were ruled out. He was discharged on anticoagulation with low molecular weight heparin for 30 days and then switched to direct oral anticoagulants for life.

## Discussion

Literature review revealed that the only other reported case of isolated PTGM and a major arterial occlusion was a 74-year-old woman with a mobile thrombus of thoracic aorta [2]. Other instances with major arterial occlusion had at least two inherited coagulopathies present concurrently. For example, a case report demonstrated bilateral superficial femoral artery thrombosis in a patient with the PTGM. However, this patient also carried a diagnosis of antiphospholipid syndrome [3].

A recent meta-analysis did not demonstrate significant association between PTGM and chronic limb ischemia secondary to atherosclerotic disease of the lower extremity. Nevertheless, the study suggested a higher prevalence of PTGM in atherosclerotic occlusive disease of the lower extremities presenting with critical limb ischemia [4]. Our patient did not have established peripheral vascular disease.

PTGM is known as a genetic variation in the prothrombin gene with G>A transition at nucleotide 20210 which leads an increase in the prothrombin levels in blood. However, studies have shown normal prothrombin fragment F1 + 2 levels in patients with PTGM and this does not support ongoing spontaneous in vivo thrombin generation. In contrast, the elevated endogenous thrombin potential has revealed that thrombotic tendency in patients with the mutation is more likely related to increased formation of thrombin once thrombin generation is triggered [5]. These finding suggests that in patients with the PTGM, an unrelated insult must first trigger the clotting cascade. Nevertheless, our patient had a major arterial thrombosis in the setting of isolated PTGM with no other identifiable triggering factor, which makes it a unique case to report.

## Conclusions

A prothrombin gene mutation has traditionally been reported to cause venous thrombosis and arterial thrombosis is suspected only when there is a pre-existing cause. This case report indicates that in patients with PTGM, arterial thrombotic events are possible complications in the absence of another cause of hypercoagulabilty.
